# Prediction complements explanation in understanding the developing brain

**DOI:** 10.1038/s41467-018-02887-9

**Published:** 2018-02-21

**Authors:** Monica D. Rosenberg, B. J. Casey, Avram J. Holmes

**Affiliations:** 1 Department of Psychology, Yale University, New Haven, CT 06520 USA; 2 Department of Psychiatry, Yale University, New Haven, CT 06511 USA

## Abstract

A central aim of human neuroscience is understanding the neurobiology of cognition and behavior. Although we have made significant progress towards this goal, reliance on group-level studies of the developed adult brain has limited our ability to explain population variability and developmental changes in neural circuitry and behavior. In this review, we suggest that predictive modeling, a method for predicting individual differences in behavior from brain features, can complement descriptive approaches and provide new ways to account for this variability. Highlighting the outsized scientific and clinical benefits of prediction in developmental populations including adolescence, we show that predictive brain-based models are already providing new insights on adolescent-specific risk-related behaviors. Together with large-scale developmental neuroimaging datasets and complementary analytic approaches, predictive modeling affords us the opportunity and obligation to identify novel treatment targets and individually tailor the course of interventions for developmental psychopathologies that impact so many young people today.

## Abstract (web summary)

This review summarizes how predictive modeling, a method that uses brain features to predict individual differences in behavior, is used to understand developmental periods. Rosenberg et al focus specifically on adolescence and examples of characteristic adolescent behaviors such as risk-taking.

## Introduction

Understanding how the brain gives rise to cognition and behavior is a fundamental goal of human neuroscience. Scientists, philosophers, and statisticians have long debated the nature of understanding, but tend to agree that there are two routes to achieving it: explanation and prediction^1–4^. Despite the historical dominance of explanation as a route to understanding, scientists and philosophers of science have emphasized the importance of both these approaches^5–8^. As noted by the philosopher Heather Douglas, “explanation and prediction are best understood in light of each other and thus … should not be viewed as competing goals but rather as two goals wherein the achievement of one should facilitate the achievement of the other”^7^.

Foundational cross-species research has made significant progress on the path towards neuroscientific explanation. Researchers have described neural bases of cognition, characterizing how patterns of brain organization from neural circuits to functional networks relate to behavior and psychopathology^9–14^. Although this work has traditionally taken a cross-sectional, group-level approach to studying the developed adult brain, there is growing consensus that comprehensive models in neuroscience must account for the facts that neural phenotypes and behavior vary widely across the population and change over time within individuals^15–18^.

The road to prediction is less traveled. Recently, however, the use of machine-learning methods to predict behavior from brain measures has become increasingly common, due in part to the emergence of large data sets and new analytic and computational tools^19^. Representing a critical avenue to understanding, these approaches provide new ways to account for developmental changes in behavior, dynamic brain systems, and associated individual differences while offering statistical rigor^8,20^ and clinical and translational benefits for personalized medicine and education^5,6^.

Although much predictive modeling research has focused on adults, forecasting outcomes in childhood and adolescence presents unique opportunities for scientific discovery and clinical application. First and foremost, biological and statistical models that account for developmental change are necessary for truly understanding how neural circuits emerge to give rise to cognition and behavior. Predicting behavior from brain features during development represents initial progress towards this goal, and predicting future outcomes from past developmental changes represents an important next step. Predictive models of current and future behavior may be especially beneficial in adolescence, a developmental period of rapid social, emotional, psychological, and physical change characterized by mental and physical health vulnerability, but also opportunities for growth and intervention^21^.

In this forward-looking review, we highlight how predictive modeling in developmental neuroscience can account for developmental trajectories in behavior, dynamic brain systems, and individual differences in both. After discussing these concepts in the context of adolescence, we introduce predictive modeling and its applications in developmental populations. Using adolescence as a case study, we address two complementary questions. First, how can prediction inform models of risk taking, a phenotype that is strikingly elevated in adolescence? Second, how can considering adolescence inform predictive modeling techniques and motivate future research? We conclude by emphasizing the importance of approaches that predict current and future behavior from developmental trajectories of brain structure and function. In doing so, we discuss how these methods complement and extend ongoing research on the neurodevelopmental processes that underlie the emergence and disruption of cognition and behavior.

## Changes in behavior and brain systems across adolescence

Human abilities and behavior change dramatically across the lifespan, emerging over development from the dynamic interplay between genes and experience. Developmental changes reflect neurobiological constraints shaped by evolution to meet the unique challenges of each stage of life, including adolescence^22^. That evolutionary pressures have presumably tailored adolescent behavior to facilitate the transition to independence, however, is frequently overlooked. Instead, adolescents, whose behavior is sometimes judged as immature relative to their physical development, are often considered impaired mini-adults^22–24^. In the following section, we emphasize the importance of considering developmental changes, dynamic brain systems that unfold over time, and interindividual variability when seeking to establish descriptive and predictive models of behavior.

### Developmental trajectories in behavior

When we think of the prototypical adolescent (or recall our own teenage years), a number of quintessential traits may come to mind. We might consider (or remember) risky behaviors like dangerous driving, illegal substance use, irresponsible sexual behavior; preoccupations with peer groups and social hierarchies; an uptick in feelings of anxiety; and heated conflicts with parents, teachers, or other well-meaning figures of authority.

Epidemiological studies confirm that our stereotypes largely reflect typical adolescent behavior. Adolescents are more likely to be injured or killed in motor vehicle accidents, contract sexually transmitted infections, engage in criminal activity, and experiment with drugs than children or adults^22,23^. These behaviors are thought to stem from adolescents’ increased sensation-seeking^25,26^ and reward-sensitivity^27,28^, as well as decreased self-control^29^ and emotional regulation abilities, especially in social contexts^30–34^. The prevalence of anxiety disorders also peaks in adolescence, underscoring this developmental period as a time of both vulnerability and opportunity for intervention^21^.

Given that risky behavior during development has potentially dire consequences, why does it persist across generations and species^35^? Fear learning provides a useful example of potential evolutionary benefits of seemingly costly behavior during adolescence^36,37^. Across altricial species, whose young rely on parents for survival, fear learning is suppressed in early infancy, presumably to ensure caregiver attachment even in cases of neglect or abuse^38,39^. In adolescence, fear of previously aversive environmental contexts is diminished whereas fear of previously aversive cues (i.e., conditioned stimuli) is amplified, a pattern that may facilitate exploration and independence but also safety from immediate threat^40,41^. Importantly, these survival-relevant behaviors do not develop in a vacuum. Rather, common genetic variations^42^ and early life stressors^43–45^ affect how fear learning changes over time, influencing risk for negative outcomes such as anxiety disorders^42^. Just as developmental changes in fear learning confer both costs and benefits, changes in risk taking during adolescence are advantageous at the group level but in some contexts may be detrimental for the individual^24^. The same is likely true for other processes following their own nonlinear trajectories across development, including decision making^46^, reward learning^47^, and sensitivity to motivational^48^, appetitive, and aversive cues^49,50^.

Although stereotypes can paint teenagers in an unflattering light, recognizing that adolescent behaviors are single points along broader, evolutionarily advantageous developmental trajectories provides a more accurate, nuanced (and perhaps sympathetic) picture. Analytic perspectives that consider behavioral shifts during the transitions into and out of adolescence, as well as their differential expression across environmental and social contexts, are necessary for understanding how the brain gives rise to behavior over time.

### Dynamic brain systems

The nonlinear behavioral trajectories observed across adolescence emerge from a cascade of hierarchical changes in brain circuitry that were themselves shaped over the course of our evolutionary lineage^22^. First to mature are connections within subcortical-limbic circuits, followed by connections between cerebral cortex and subcortical-limbic circuits, and, finally, connections across cortex^51,52^.

Evidence for this developmental cascade comes from observations of earlier changes in synaptic morphology and neurotransmitter systems in subcortical relative to cortical regions and an earlier plateau in synaptic formation and subsequent pruning in unimodal sensory, motor, and subcortical regions relative to multimodal association areas^53,54^. These processes likely contribute to gray matter volume and cortical thickness changes observed during adolescence and early adulthood^55–58^ that end in the association cortices^59–61^. Selective degradation of excitatory synapses also affects the excitatory-inhibitory balance across cortex, an equilibrium related to shifts in cognitive abilities and behavior^51,62^. The relative decrease in prefrontal behavioral regulation is reflected in changes in dopamine receptor density, related to learning and reward prediction, that peak in the striatum during adolescence but not until early adulthood in the prefrontal cortex^63–65^.

Structural and functional brain connections follow similar patterns of development, providing additional evidence for a hierarchically emerging system first dominated by mature subcortical circuits and then balanced through interactions with late-maturing prefrontal systems^51,66^. As early as 1920, Flechsig’s histological studies revealed protracted myelin development in association cortex^67,68^. Reflecting this property of brain maturation, diffusion tensor imaging studies, which measure water diffusion modulated in part by axon myelination, suggest that the development of posterior cortical-subcortical tracts precedes that of fronto-subcortical tracts supporting top-down control of behavior^69–72^. Functional brain connectivity studies support these results, observing a general pattern of weakening short-range functional connections followed by strengthening long-range cortical connections across adolescence^73–76^.

Altogether, this work provides evidence for the progressive development of connectivity within and between subcortical and cortical brain regions, and offers a plausible neurobiological account of nonlinear trajectories in risk-related processes such as self-control, reward sensitivity, and emotion regulation. Emotional reactivity, for example, may arise from the early dominance of subcortical over cortical circuitry, later waning as cortical-subcortical circuits related to top-down control, and then cortical circuits involved in processes such as cognitive reappraisal, mature during adolescence and adulthood^52^. More broadly, these findings highlight how approaching the study of adolescence from a dynamic, multimodal, circuit-based perspective (rather than a view that focuses on snapshots of individual brain regions in isolation) can inform our understanding of self-regulation and risk-taking behavior during development^51,52^.

### Individual differences

Although neurobiology and behavior tend to unfold in predictable ways across development, significant individual differences lie atop this scaffolding. This variability applies not only to an adolescent’s current behavioral and neural characteristics, but also to their past and future phenotypes. That is, while one stereotype of adolescents is that they engage in risky behaviors such as binge drinking, there are plenty of young people who do not fit this mold. Even among adolescents who drink excessively, some may go on to develop substance use disorders, while others may never progress to disordered drinking.

Despite recognizing these individual differences, in research, clinical, legal, and educational practice, we often treat variance around average behavioral and neural phenotypes and trajectories as noise, or collapse it into discrete categories (e.g., patients vs. controls, adults vs. minors, etc.). Although these groups can be useful in practice, they do not necessarily represent biologically plausible or informative qualitative distinctions. Instead, approaches that characterize the normative trajectories of dimensional behavioral and neural phenotypes, and investigate how genetics and experience affect the timing and shape of these curves, are necessary for understanding how these processes unfold in development^18,66,77^. In addition to informing models in basic science, individual differences approaches can provide clinically applicable insight into the factors that confer risk for and resilience to psychopathology^44^ and guide personalized treatments^21^.

## Predictive modeling and its importance in developmental neuroscience

**Fig. 1:**
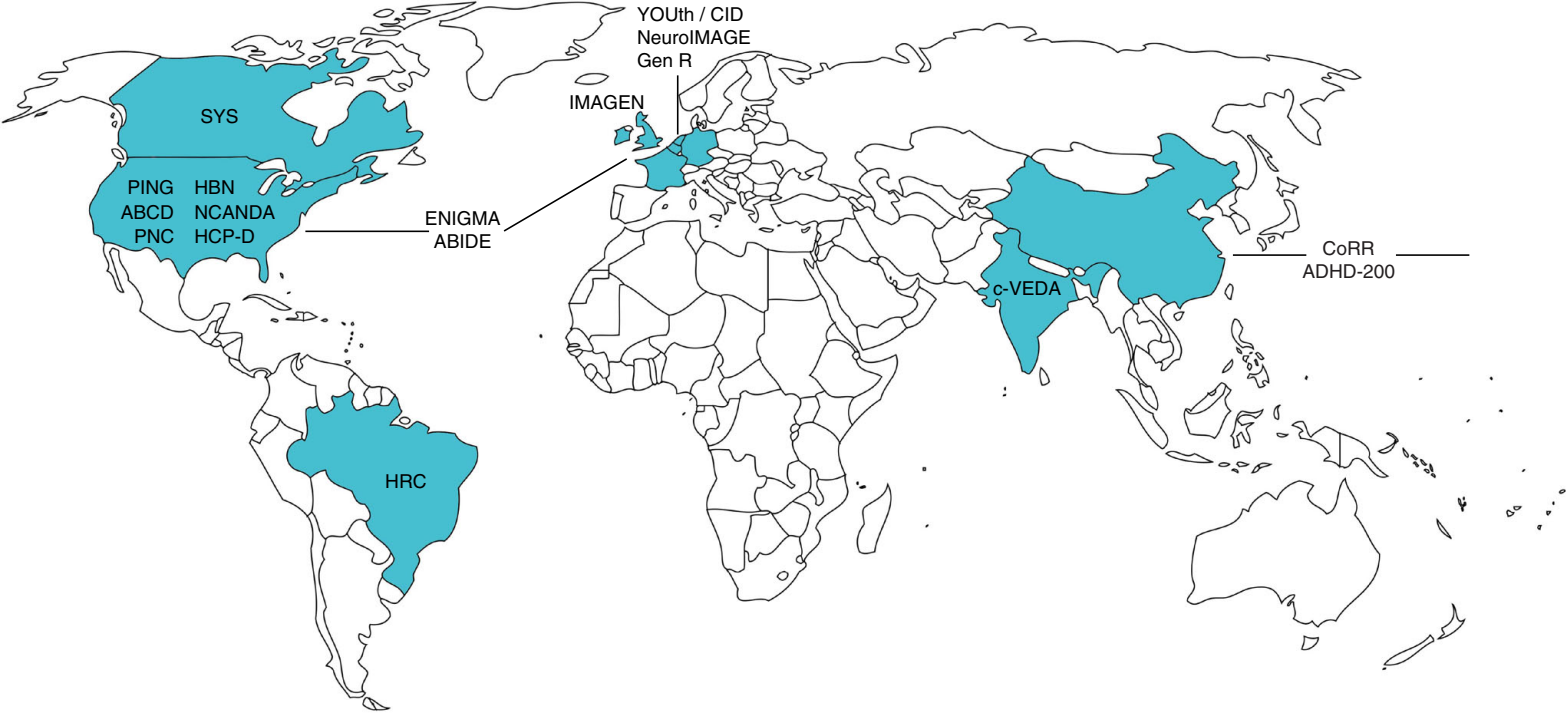
Existing, ongoing, or planned data sets including structural and/or functional neuroimaging data from ~500 or more children or adolescents. These data sets, which represent both prospective and retrospective samples, include the Adolescent Brain Cognitive Development study^83^ (ABCD; USA), Healthy Brain Network^82^ (HBN; USA), Lifespan Human Connectome Project Development^80^ (HCP-D; USA), National Consortium on Alcohol and NeuroDevelopment in Adolescence^149^ (NCANDA; USA), Pediatric Imaging, Neurocognition, and Genetics study^150^ (PING; USA), Philadelphia Neurodevelopmental Cohort^151^ (PNC; USA), Saguenay Youth Study^152^ (SYS; Canada), High Risk Cohort Study for the Development of Childhood Psychiatric Disorders^153^ (HRC; Brazil), Autism Brain Imaging Data Exchange^81^ (ABIDE; USA, Germany, Ireland, Belgium, Netherlands), Enhancing NeuroImaging Genetics through Meta-Analysis^154^ (ENIGMA; worldwide), IMAGEN^79^ (England, Ireland, France, Germany), Dutch YOUth cohort (part of the Consortium on Individual Development, or CID; Netherlands), Generation R Study^155^ (Gen R; Netherlands), NeuroIMAGE^156^ (follow-up of the Dutch arm of the International Multicenter ADHD Genetics, or IMAGE, project; Netherlands), Consortium on Vulnerability to Externalizing Disorders and Addictions (c-VEDA; UK, India), Consortium for Reliability and Reproducibility^157^ (CoRR; China, USA, Canada, Germany), and ADHD-200^108^ (USA, China). Although samples are distributed across the globe, African, Middle Eastern, South Asian, Oceanian, and Central and South American populations are underrepresented. Data collection efforts in these regions and others will be important for ensuring diverse, representative samples that will allow researchers to uncover general principles of the developing brain. (Map outline courtesy of Wikimedia user ‘Loadfile’ and is licensed under a CC BY SA 3.0 license)

Studying developmental trajectories, dynamic brain systems, and individual differences is becoming increasingly feasible with the rise of high-throughput data collection efforts^78^. Longitudinal and cross-sectional samples of neuroimaging data from children and adolescents, such as the IMAGEN study^79^, Lifespan Human Connectome Project Development^80^, Brain Imaging Data Exchange^81^, Healthy Brain Network Biobank^82^, and Adolescence Brain Cognitive Development Study^83^, have accelerated advances in basic and applied neuroscience (Fig. 1). Collaborative initiatives have also helped democratize data access, improve statistical power, and facilitate transparent, reproducible research. The unique challenges posed by large-scale imaging samples, such as how to perform adequate quality control^84^, account for scanner and site effects^85,86^, and disentangle meaningful explanatory power from statistical significance^87^, are also motivating the development of new data collection^88^, preprocessing^84^, and analytic^89^ approaches.

**Fig. 2:**
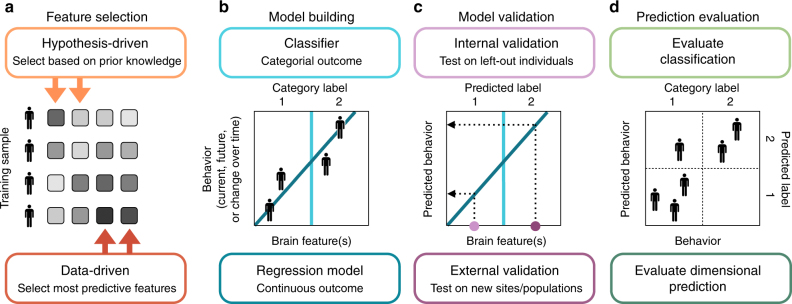
Schema of key concepts in predicting individual differences in behavior from brain features. **a** Feature selection. Feature selection techniques fall into two broad categories: hypothesis-driven (top-down) and data-driven (bottom up) approaches. **b** Model building. Machine-learning algorithms can be used to predict categorical measures, such as clinical diagnoses, or dimensional measures, such as task performance or symptom severity. Here, the dark blue line shows the relationship between a single hypothetical brain feature and a behavioral score. The light blue line illustrates a classifier that divides individuals into categories based on this brain feature. (Note that, unlike in this condensed visualization, behavioral scores are typically related to category labels.) **c** Model validation. Predictive models are evaluated on previously unseen data—either left-out individuals from the initial data set (internal validation) or individuals from a completely new sample (external validation). **d** Prediction evaluation. Continuous predictions (bottom and left axes) are evaluated by comparing observed and predicted behavioral measures, e.g., with correlation or mean-squared error. Categorical predictions (top and right axes) are evaluated with percent correct; binary predictions can be assessed with sensitivity and specificity and/or positive and negative predictive value

Large neuroimaging data sets are not only advancing understanding of how brain features relate to behavior at the group level, but are also renewing focus on the individual. Although cognitive and developmental neuroscientists have long been interested in interindividual differences in abilities and behavior, traditional experiments have focused on tens, rather than hundreds or thousands, of participants. These small samples, with tightly controlled demographics and circumscribed behavioral phenotypes, are not always conducive to studying population variability. Larger samples that capture a broad range of phenotypes provide opportunities not only to describe brain–behavior relationships, but to predict behavior from brain features at the level of single individuals^90,91^. In this vein, researchers are searching for neuromarkers, or brain features that predict behavior, clinical symptoms, risk for or resilience against illness, or treatment response^5,6,92^. The pursuit of generalizable neuromarkers goes hand-in-hand with predictive modeling, a technique that leverages brain–behavior relationships to predict outcomes in novel individuals (Box 1 and Fig. 2).

The statistician George Box famously claimed that “all models are wrong but some are useful”^93^. Models that predict outcomes from previously unseen observations can be especially useful for both scientific discovery and clinical decision-making^5,6^. From a basic science perspective, predicting brain–behavior relationships at the level of single subjects represents progress towards understanding how individual differences in brain features relate to individual differences in cognition and behavior^94^. In addition, because predictive models are by definition validated on independent data, they can help foster robust, reproducible discoveries.

The benefits of individualized predictions of current and future behavior are especially pronounced in developmental populations including adolescents. Because behavior and psychopathology are best viewed as the result of developmental processes that unfold across the lifespan^21,66^, characterizing individual arcs in brain–behavior relationships over time can move us even closer to understanding targets for change. Addressing the unique challenges presented by prediction in adolescence, including the complex dynamics linking neurobiological, behavioral, and environmental change, can also help us better model periods such as prenatal development, infancy, aging, and illness course.

Predictive models may not only contribute to progress in basic developmental neuroscience, but may also have implications for education, mental health, and legal policy. For example, early predictions of behavioral impairments could facilitate earlier treatments and improved health or educational outcomes^6^. Prediction can also inform pressing policy questions, such as characterizing the maturity of a particular individual in specific contexts to inform whether they should be treated as an adult in the justice system^95,96^. Thus, although machine-learning models of behavior in development may be “wrong” in the sense that they (necessarily) simplify complex neurobiological systems, they are useful in that they can inform theories of how cognition and behavior emerge from dynamic brain systems and speak to general educational, medical, and social policies.

## Predictive modeling and risk preference

One of the most common reprimands of wayward youths is: “Act your age!” The phrase — immortalized in the English-language idiom “act your age, not your shoe size” — is so ubiquitous that it even makes an appearance in song lyrics from the musical artist Prince. Its sentiment, however, is not straightforward. What does it mean for an adolescent or young adult to act his or her age? What counts as typical adolescent behavior? One possibility is that “act your age” means, “make the most responsible decision you have the capacity to make”. What this entreaty fails to recognize, however, is that there is a discrepancy between how responsibly adolescents and young adults can act in nonsocial, unemotional situations relative to social or emotionally charged contexts^23,96^.

**Fig. 3:**
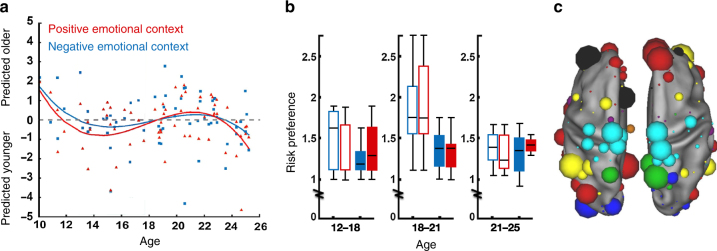
Adolescents’ functional connectivity patterns look younger in emotional contexts. Adapted with permission from Rudolph et al.^97^. **a** Chronological age is plotted against age predicted from functional connectivity patterns observed in positive and negative emotional contexts. Individual points (participants) are fit with polynomial curves. On average, adolescents are predicted younger in emotional contexts. **b** Adolescents (age 12–18; numerical difference) and young adults (age 18–21; *p < *0.1 in negative emotional contexts and *p* < 0.05 in positive emotional contexts) who are predicted younger in emotional contexts tend to show greater risk preference. This trend is most pronounced in young adulthood. Open bars represent individuals predicted younger in emotional contexts, and filled bars represent individuals predicted older. Red bars show participants grouped by age predictions in positive emotional contexts; blue bars show participants grouped by age predictions in negative emotional contexts. **c** Functional network nodes, scaled by their importance in the age-prediction model, are grouped into the following functional networks defined previously^13^: default mode (red), dorsal attention (green), frontoparietal (yellow), salience (black), cingulo-opercular (purple), visual (blue), subcortical (orange), and ventral attention (teal).

Recent work from Rudolph and colleagues^97^ used predictive modeling to identify the neural basis of this phenomenon, asking whether functional brain organization looks less mature in emotional contexts, and whether this effect relates to individual differences in risky behavior. To this end, the authors calculated functional connectivity patterns from fMRI data collected while 212 individuals aged 10–25 performed a go/no-go task in neutral and emotional contexts. During emotional contexts, participants anticipated an aversive noise or a reward; during neutral contexts there was no anticipation of noise or reward. Using partial least squares regression and 10-fold cross-validation, the authors first built a model to predict chronological age from functional connectivity patterns observed in the neutral context, and then applied the same model to connectivity observed during the emotion manipulation. They found that a prediction made from an individual’s neutral context pattern (their “neutral brain age”) was closer to their chronological age than a prediction made from their emotional context pattern (their “emotional brain age”). Further, both predictions tended to be younger than chronological age in teens. Interestingly, there was a trend such that adolescents were more likely to look younger in emotional relative to neutral contexts, but young adults who showed this pattern had greater risk preference and lower risk perception^97^ (Fig. 3). These findings illustrate the power of predictive modeling in delineating dynamic developmental changes and individual differences in risk taking behaviors.

In addition to helping explain why adolescents may not “act their age” under emotional arousal, the Rudolph et al. findings raise two notable points about predictive brain-based models in general.

First, when the model of chronological age was wrong, it was wrong in interesting ways: A young adult incorrectly predicted younger in an emotional context was more likely to show a “risky phenotype” than an individual incorrectly predicted older. Thus, in some cases, model errors may be as informative as model successes in unraveling the brain bases of individual differences in behavior.

Second, this model—along with many in cognitive and developmental neuroscience—predicts outcomes from functional connectivity data. Given that functional connectivity patterns can be affected by cognitive state^94^, such models may not generalize across contexts as well as models based on state-independent features such as structural connectivity. (There is evidence, however, that functional connectome-based models generalize across task-engaged and resting states to predict abilities such as attention^98^.) Thus, researchers hoping to build an age-prediction model with optimal predictive power and generalizability may consider including structural features that may capture more “trait”-related than state-related variance as predictors (see the section entitled “*Include multimodal predictors*”).

Finally, it is important to note that although here maturity was assessed with a single number—akin to the difference between an individual’s functional connectivity pattern and the age-typical pattern—maturity does not lie on one continuum from “less” (in emotional states) to “more” (in unemotional states). Rather, temporal differences in the fine-tuning of interacting neural systems with age and experience impact behavioral phenotypes differently across development and vary across individuals and contexts^52^. For example, Rudolph and colleagues show that, on average, adolescents’ functional connectivity profiles look younger in emotional contexts, and that young adults who maintain this profile show riskier choices. This work suggests that future studies can characterize each individual’s unique multivariate maturational profile, that is, the age-typicality of both their trait- and state-dependent neural phenotypes.

## The road ahead

Just as building predictive models can inform how we understand risk taking in adolescence, studying adolescence can inform how we approach behavioral prediction. Here, motivated by predictive and descriptive models of development, we suggest eight directions for future research and highlight their importance for understanding the neurobiological basis of adolescent behavior. In particular, we encourage researchers to bridge data sets and levels of analyses to develop generalizable, trajectory-based models that predict current and future outcomes.

### Leverage multiple data sets to build and validate predictive models

Predictive models will be most theoretically and practically useful when they generalize beyond a single data set. Although historically replication and external validation samples were rare in fMRI due to cost and time constraints, open-access data sets and a growing culture of data sharing are removing barriers to access. Consider, for example, a group of investigators interested in predicting impulsivity from resting-state functional connectivity data. These researchers could download data from the Human Connectome Project^99^, model the relationship between impulsivity and functional connectivity, and then apply their model to completely independent data from the Brain Genomics Superstruct Project^100^ to evaluate its generalizability.

Training and testing predictive models with open data sets has obvious benefits. For our hypothetical investigators, downloading data may cost a fraction as much as running their own, smaller fMRI study. Open data sets also tend to offer relatively large sample sizes, capturing a wide range of behavior and allowing researchers to fit complex models^90^ and refine model parameters with nested cross-validation techniques. In addition, open samples can provide opportunities to validate models across unique behavioral measures. Although this approach can be challenging given that different-but-related measures may index similar-but-not-identical mental processes, it is a useful way to investigate whether a model is capturing individual differences in a specific performance metric or a general cognitive function. For example, imagine that researchers build a model to predict impulsivity questionnaire scores. If they apply this model to a new sample in which impulsivity is measured with task performance, predictive power will be limited by the ground-truth relationship between questionnaire scores and task performance. Successful generalization would provide additional evidence that the model is related to individual differences in impulsivity per se rather than individual differences in questionnaire scores alone. Thus, validating models in open data sets can help establish their specificity and generalizability.

This should not be taken imply that targeted studies are obsolete. Instead, experiments designed to probe specific behavioral phenotypes with carefully designed psychological tasks are crucial complements to open data analyses. Because targeted studies have greater flexibility in the participants they recruit, the behavioral measures they collect, and the tasks they administer, they can help elucidate brain–behavior relationships across populations and cognitive states. The impulsivity research group, for example, could use data from a targeted study to ask whether the same functional network that predicts impulsivity in adults emerges in development to support children’s impulse control. (In fact, they may not even need to collect their own data to do so: Relevant targeted samples may be available on data-sharing platforms such as OpenfMRI^101^.) Recent work examining the heritability of the functional connectome used a similar approach, building a model of siblingship in a locally acquired data set, and validating it in the Human Connectome Project sample^102^.

The sustained attention connectome-based predictive model is another recent example of a model validated across multiple imaging data sets^98,103–107^. This model was defined to predict individual differences in the ability to maintain focus from patterns of task-evoked and resting-state functional connectivity^103^. During fMRI, adult participants performed a challenging sustained attention task, which presumably perturbed attention-relevant neural circuitry and amplified behaviorally relevant individual differences in functional connectivity. Models defined on task-based data generalized to predict left-out participants’ task performance not only from data acquired as they were engaged in the task, but also from data collected as they simply rested. External validation with data from the ADHD-200 Consortium^108^ revealed that the same functional networks that index attention task performance in adulthood predict ADHD symptoms in childhood. Together these results suggest that a common functional architecture supports sustained attention across developmental stage (adults vs. children and adolescents), clinical population (ADHD vs. control), and cognitive state (task vs. rest)^103^.

Another targeted study provided insights into potential mechanisms of the model’s predictive networks. That is, the same sustained attention connectome-based predictive model distinguished individuals who had taken a single dose of methylphenidate (Ritalin) from controls, raising the possibility that networks reflect the expression of neurotransmitters whose extracellular concentration is modulated by methylphenidate^104^.

Although the anatomy of the sustained attention model is complex, broad trends align with previous findings and suggest new targets for intervention^103^. Functional connections between sensorimotor and cerebellar regions predict more successful sustained attention, whereas intra-cerebellar, intra-temporal, and temporal-parietal connections predict less successful attention. The participation of the cerebellum, implicated in ADHD^109,110^, provides convergent evidence of its importance for attention. Frontal and parietal regions traditionally related to attention and attention impairments do appear in the predictive networks, but they represent >35% of all connections in the model, accentuating the importance of data-driven approaches to feature selection.

In light of the sustained attention model’s out-of-sample generalizability—a recent proof-of-principle example—we are optimistic that, moving forward, a combination of high-throughput data sets, targeted experiments, and “green science” data sharing initiatives will facilitate robust, generalizable models of cognitive abilities and behavior across development.

### Develop trajectory-based models with longitudinal data

Neurobiology is inherently dynamic, and understanding any dynamic process in terms of both description and prediction requires appreciating changes over time. Atmospheric models, for example, rely on dynamical equations to predict the weather^111^, and stock forecasting models use measures of how a stock’s performance has changed in the past to predict how it will perform in the future. We often use longitudinal data to make folk psychological predictions, such as when we consider how quickly a young tennis player climbed the rankings to estimate her shot at winning Wimbledon, or use what we know about a friend’s recent stress levels to predict how he will react in an emotional situation.

**Fig. 4:**
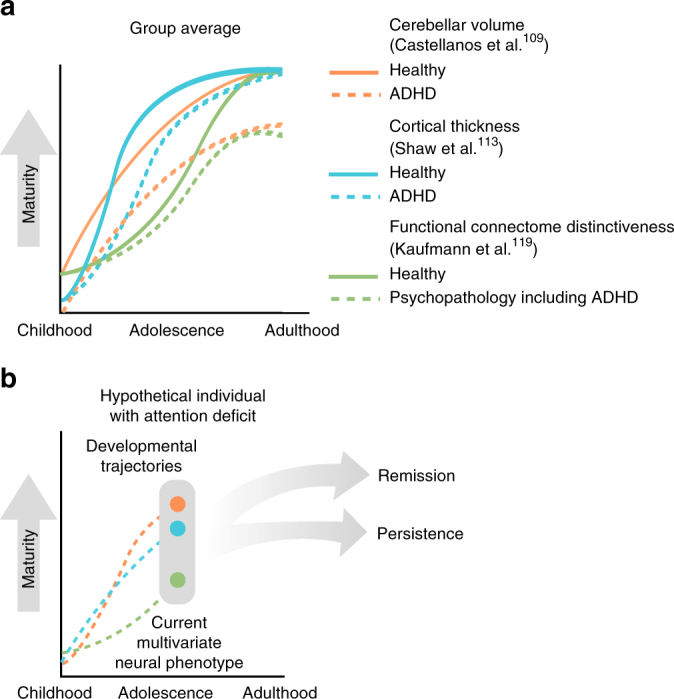
**a** Developmental changes in cerebellar volume, cortical thickness, and functional connectome distinctiveness in healthy individuals and individuals with attention deficits. Curves are based on data from refs. ^109,113,119^. **b** Developmental changes in a hypothetical adolescent with attention deficits. A model trained to use the developmental trajectories of multiple brain measures to predict future outcomes may best characterize whether this individual’s deficits will improve, persist, or worsen. These predictions may have implications for future treatment or cessation of treatment

Models that predict behavior from brain features can also benefit from longitudinal measures. Consider again the case of attention deficits. Pioneering work applied growth–curve models to cross-sectional and longitudinal data to establish delays in cortical thickness and brain surface area maturation^112,113^, as well as a down-shifted trajectory of cerebellar growth^109^ in children and adolescents with ADHD (Fig. 4; but see refs. ^114–117^ for methodological considerations related to effects of head motion). Recent work also suggests that the age-typicality of a child’s or adolescent’s functional connectivity patterns is related to their psychiatric symptoms, including attention deficits^118,119^. In other words, children and adolescents with attention deficits show delayed maturational patterns of cortical thickness and functional connectivity on average, and single snapshots of functional connectivity predict single snapshots of attentional abilities in novel individuals. It follows that a teenager’s unique trajectory of functional connectivity and cortical thickness development may provide more nuanced information about his or her attentional abilities, predicting not only deficit severity, but also perhaps symptom persistence or abatement. Developmental neuroscientists pursuing trajectory-based predictive models can take advantage of large longitudinal samples such as IMAGEN or the open-access ABCD collection effort, and of biostatistical techniques developed to predict clinical outcomes from longitudinal biomarkers^120–123^.

In addition to potentially increasing predictive power, individualized trajectory-based models can inform theories of how neural phenotypes give rise to typical and atypical development. For example, although we know that delayed cortical maturation trajectories characterize ADHD patients at the group level^113^, it is not yet known whether a delayed trajectory confers risk for attention deficits at the level of individual subjects. Extending similar group-level findings to the level of individual subjects can enhance the clinical utility of research findings and inform novel interventions^44^.

### Predict future outcomes

Models that predict current behavioral tendencies are technically postdictive in that they make retrospective, rather than prospective, predictions. Although these models can inform relationships between neural and behavioral phenotypes, models that predict future outcomes may be most useful in clinical and translational contexts, allowing for earlier intervention, treatment, or cessation of treatment.

Recent work demonstrates that models that make future forecasts are possible in the context of development. For example, Whelan and colleagues^124^ modeled neural and psychological profiles of alcohol misuse before its onset in adolescence. Using measures of brain structure and function, personality, cognitive abilities, environmental factors, life experiences, and genetic variants, the authors built a model that distinguished adolescents who go on to binge drink from those who do not. New findings suggest that brain features can predict clinically relevant outcomes even earlier in development: cortical surface area and functional connectivity observed at 6–12 months, for example, predict autism diagnosis at age two^125,126^.

In addition to models that predict the onset of clinical symptoms or risky behavior, models that predict improvements in clinical outcomes can help identify resilience factors for psychopathology. Recently, Plitt and colleagues^127^ used functional connectivity patterns to predict improvements in adolescents’ and young adults’ autism symptoms. They found that functional connectivity in the salience, default mode, and frontoparietal networks, implicated in attention and goal-directed cognition^11,12^, predicted symptom changes over time, even when accounting for age, IQ, baseline symptoms, and follow-up latency. Hoeft and colleagues^128^ also showed that prefrontal activity and right superior longitudinal fasciculus fractional anisotropy, but not reading or language test scores, predicted which children with dyslexia would show reading skill improvement over the course of 2.5 years. Functional and structural measures, therefore, can predict not only the onset of clinical symptoms, but also the abatement. In addition, brain features can predict future outcomes over and above behavioral measures alone—an important check when evaluating the utility of predictive models.

In the future, trajectory-based approaches may better characterize not just where a child or adolescent has been or is currently, but where he or she is going. For example, models that use a child’s developmental growth curve (e.g., precocious, delayed, deviant, regressive, or resilient^18,77,^) to predict the persistence or worsening of clinical symptoms could have implications for treatment. Such models may also have implications for the cessation of treatment. A child with attentional impairments but a resilient developmental trajectory for ADHD could, for example, be titrated off of medication sooner than otherwise possible (Fig. 4).

### Include multimodal predictors

Models in human neuroscience often focus on a single type of brain feature, such as functional connectivity, to predict behavior. Although this approach is useful for targeting specific neural mechanisms, constraining a model’s feature space to a single modality may limit predictive power. Open-access data sets including a variety of scan types (e.g., T1-weighted, T2-weighted, proton density, T2-FLAIR, DTI, BOLD) facilitate the construction of models incorporating a range of features (e.g., myelination patterns, structural connectivity, task-based and resting-state functional connectivity) to maximize predictive power and uncover the unique contributions of different neural systems to current and future behavior.

Researchers have demonstrated that including multiple feature classes improves individualized predictions in development not just in theory, but also in practice. For example, in the first-ever example of predictive modeling in developmental neuroscience, Dosenbach and colleagues^129^ asked whether resting-state functional connectivity patterns can predict an individual subject’s chronological age. Using data from 238 individuals aged 7–30, they trained models to predict categorical (child vs. adult) and dimensional (chronological age) measures of maturity. A support vector machine classifier correctly predicted whether an individual was a child or an adult 91% of the time, and a support vector regression algorithm accounted for 55% of the variance in chronological age. Motivated to find the unique contribution of multiple neuroanatomical features to age predictions, Brown and colleagues^130^ built a model based on structural features that accounted for 92% of the variance in age in a sample of 885 individuals aged 3–20. Interestingly, different features contributed to model performance at different ages: Whereas T2 signal intensity in subcortical ROIs was most diagnostic of age in childhood, fiber tract diffusivity and subcortical structure volume were most informative in adolescence, and ROI diffusivity was most informative in adulthood^130^. Franke et al.^131^ additionally found that predictions of a “brain age” model based on multiple neuroanatomical features were significantly younger for adolescents born preterm than full term. Although the variance explained by functional connectivity and neuroanatomy cannot be directly compared given differences in methodology and participant samples across studies, multimodal approaches may capture more variance in individual differences than do unimodal ones.

Black box models that use an assortment of brain features to predict outcomes may not necessarily provide interpretable links between neurobiology and behavior. How can researchers unravel the unique contributions of different feature classes to individual differences? In modeling alcohol misuse in adolescence, Whelan and colleagues provide one example of how this may be achieved. To start, their model of future binge drinking included neural, behavioral, lifestyle, and genetic factors. They then systematically removed each feature class from the model to isolate its contribution to predictive power. The approach revealed that life history, personality, and brain variables were most uniquely predictive^124^. Other approaches, such as dimensionality reduction strategies and penalized regression methods, can also help eliminate redundant predictor variables and identify those most tightly coupled with individual differences in behavior.

Although hypothesis-driven modeling approaches that use a single feature or feature type to predict outcomes can advance knowledge about the neurobiological bases of behavior, multivariate models that incorporate both structural and functional features may improve prediction accuracy and offer converging insights (e.g., Fig. 4).

### Continue to focus on dimensional outcomes

Centuries of observation and research tell us that cognitive abilities and behavior vary along a continuum at every stage of life. Consider the example of impulsivity: Although variability in impulsivity can be captured, to some degree, with a categorical measure like an ADHD diagnosis, it may be better quantified with dimensional measures such as symptom severity or impulse-control task performance.

When characterizing brain–behavior relationships in development, the costs and benefits of using categorical vs. dimensional measures should be carefully considered. Although categorical labels align with the current diagnostic system in clinical medicine and can help make results easier to interpret and present, dichotomizing continuous variables can reduce statistical power, obscure nonlinear relationships between variables and outcomes, and increase the risk of false positive results^132,133^. In addition, categorical models are often built on balanced samples of patients and control participants to avoid biased predictions, but this ratio rarely reflects real-world illness prevalence. Thus, reported measures of a model’s sensitivity and specificity may exaggerate its translational utility, and positive and negative predictive values may be more useful measures of performance^134^ (see Box 1). Dimensional measures may better characterize the full range of behavior and clinically relevant outcomes, especially in development, when small differences in behavioral or neural phenotypes can have important implications for treatment.

Although dimensional measures are frequently used to characterize individual differences in cognitive and developmental neuroscience and psychology^18,135^, such approaches are infrequent in predictive modeling^5^. Models that predict chronological age^97,129–131^, fluid intelligence^136–138^, attention^103,118,139^, and improvements in math skills^140^ and autism symptoms^127^, however, demonstrate that such approaches are powerful ways to identify robust transdiagnostic biomarkers of abilities and behavior.

Looking ahead, modeling approaches that consider multiple dimensional approaches at once, or those that identify latent distributions from which behavior emerges, may help delineate subtypes of clinical disorders and improve outcome predictions and treatment^17^. Regression models that predict dimensional outcomes and consider subgroups that make up heterogeneous patient populations will also continue to be valuable complements to classifiers that predict group membership.

### Establish boundary conditions

Given that brain structure and function change dramatically across development, models trained in one developmental period (e.g., adulthood) should not always generalize to others (e.g., adolescence). Rather, upper bounds on models’ predictive power will be influenced by the reliability of the brain and behavioral measures, their stability across development, and the developmental trajectories of the underlying neurobiology.

Testing models across different developmental periods can help identify critical change points in the relationship between neurobiological processes and behavior. As a concrete example, the sustained attention connectome-based predictive model introduced earlier generalized from an adult to a developmental sample^98,103^. The model, however, did not perform equally well in all age groups. Although predictions were significantly related to ADHD symptoms in children 8–9 (*n* = 30), 10–11 (*n* = 28), and 12–13 (*n* = 41), they did not reach significance in adolescents 14–16 (*n* = 14; unpublished results). Although certainly not conclusive given the exploratory nature of this analysis and the fact that predictive power is influenced by factors including sample size, data quality, and group variance in ADHD scores, this outcome motivates future research by tentatively suggesting that the functional architecture of attention in adolescence may differ from that in childhood and adulthood. These findings also underscore the importance of understanding the nonlinear expression of dynamic and hierarchical changes in brain features and behavior with development.

Moving forward, it will be important to tailor predictive models to particular scientific questions and/or practical goals. For example, models trained on one developmental period and tested on another can inform questions about common functional mechanisms, whereas models trained on a range of age groups may better characterize trajectories in brain–behavior relationships and offer greater predictive power across the lifespan. Further, it is important to keep in mind that because children and adolescents are not simply “little adults” in terms of either neurocircuitry or behavior, predictions in these populations will likely often rely on development-specific models rather than models defined in adults and applied to developmental data. Future work testing whether models are valid across developmental stages, clinical populations, and cognitive or affective states can provide additional insight into the scope of their generalizability.

### Bridge statistical predictability and biological plausibility

Predictive modeling in developmental neuroscience has two parallel goals: to discover how the brain gives rise to behavior across development, and to identify practically useful neuromarkers of behavior and clinically relevant outcomes. It is not always obvious, however, how models that achieve the second goal can help make progress toward the first. Instead, predictive models are sometimes considered opaque “black boxes” far removed from biology and uninformative about the neural circuits supporting behavior. Some models are more susceptible to this concern than others. Supekar and colleagues^140^, for example, used hippocampal volume and functional connectivity to predict children’s response to math tutoring. In doing so, they provide clear evidence of the role of learning- and memory-relevant brain regions in math skill improvements. On the other hand, models that use deep neural networks to generate predictions may sometimes preclude easy (linguistic) interpretations of relationships between predictors and outcomes. As bigger data sets and more sophisticated algorithms result in greater and greater predictive power, it will be important for researchers to keep the first goal of modeling—advances in basic science—in their sights.

Large-scale data sets that include behavioral, neuroimaging, and genetic data provide exciting opportunities for researchers to explore the biological plausibility of predictive models. To illustrate the promise of approaches that link levels of analysis, let’s return to the hypothetical research group interested in impulsivity. Imagine that the research team identifies a pattern of functional connectivity that predicts impulsivity across individuals. A subsequent issue of clear importance is the extent to which this network reflects the underlying function of molecular-genetic mechanisms. To get initial traction on this question, the researchers could ask whether this network is heritable, or whether structural genetic variants predict its function, which in turn predicts impulsive behavior. They could also pursue cross-species work, asking whether important model features map on to known anatomical circuits, or whether hypothetical genes that impact network function in humans affect behavior in rodents. Thus, approaches that combine data sets to bridge levels of analysis and link genotype, neural phenotype, and behavior across development may suggest new etiological hypotheses and possible treatment targets of cognitive function and dysfunction.

### Acknowledge limitations to advance understanding

Enthusiasm for large neuroimaging data sets and individualized predictions should be coupled with realistic assessments of potential pitfalls related to methodology, interpretation, and implementation. Carefully considering these limitations, researchers are already beginning to develop new analytic approaches and field-wide standards to address them^84,87,89,141,142^.

Methodological pitfalls can erode the impact of predictive models. For example, because head motion introduces significant confounds in both structural and functional imaging data, especially in developmental populations^115,143^, up-to-date data-collection and preprocessing techniques are necessary for ensuring that predictions do not rely on motion-induced artifacts. In addition, just as descriptive models may reflect sample noise, predictive models may be overfit to training data. Although nested cross-validation techniques can help protect against overfitting, external validation is critical for testing model generalizability (Box 1). Finally, it is important that methodological choices be tailored to research goals. For example, is the goal to predict current phenotypes or future change? To make absolute predictions (e.g., that a child will grow to be six feet tall) or relative ones (that he or she will be in the 95th percentile for height)? To predict behavior from functional brain features observed during task engagement, or to test whether a cognitive process can be measured in the absence of an explicit task^116^? To maximize subgroup-level accuracy, or population-level generalizability? To prioritize statistical predictability, biological plausibility, or feature weight interpretability? Mismatches between study methods and goals can undermine the usefulness of predictive models.

Working with large data sets also poses several challenges to interpretation. For example, when samples are large enough, brain–behavior relationships with even tiny effects sizes may reach statistical significance. Although such effects may be interesting from a basic science perspective if robust to noise and replicable, they are unlikely to offer much clinical or practical benefit in the near term^87^. In addition, although large data sets may be more representative of the general population than smaller samples, they may offer a “false sense of security” since they can still suffer from selection bias and skewed demographics^144^ and are not equally distributed across the globe (See Fig. 1). The careful evaluation of both statistical and clinical significance will be important for establishing scientifically valid, practically useful models.

Although neuroimaging-based predictive models have the potential to offer significant benefits, challenges can arise when applying them in clinical contexts. Recent work has highlighted the “perilous path from publication to practice”, outlining a variety of scientific, implementation, and business-related obstacles^145^. As scientists pursue individualized predictions, close collaborations with clinicians, bioethicists, industry professionals, and regulatory bodies will be necessary for effectively translating models to real-life patient-care settings^141,146^.

Finally, as models begin to make their way from bench to bedside, it is important to consider their ethical implications, especially in the context of development. First, researchers need to keep in mind—and clearly communicate to participants, patients, and the public—that predictive models are probabilistic rather than deterministic in nature, and, just like traditional pen-and-paper tests, will never perfectly predict abilities or behavior. Predictions are thus best considered tools for scientific discovery and opportunities for informing clinical decision-making rather than portents of the future. Moving forward, the potential benefits of predictive modeling in development must be continuously evaluated in light any of potential risks to privacy or hyperbolic claims about our ability to predict the future.

## Conclusions

A rich tradition of research in human neuroimaging has made progress in explaining the neurobiology of cognition and behavior. Less attention, however, has been devoted to predicting cognitive abilities and behavior from brain features. Here we argue that predictive modeling approaches that forecast outcomes at the level of individuals are important complements to work describing brain–behavior relationships at the group level, especially in the context of adolescence.

Not only can predictive models enhance the clinical and translational utility of neuroimaging research, they can also account for critical features of behavior often overlooked in cross-sectional studies of the developed adult brain: developmental trajectories, hierarchically emerging brain systems, and individual differences in both. So far, investigating when models accurately predict behavior and when they fail to do so has illuminated potentially adolescent-specific^34^ changes in behavioral and neural phenotypes related to risk-taking and attention. Looking ahead, models that offer probabilistic insights into individuals’ current and future behavior from their past developmental brain trajectories have the potential to provide deep insights into human brain development and function in both health and disease.

### Box 1 Predictive modeling

Whereas descriptive modeling is the process of learning associations between features and outcomes, predictive modeling leverages these relationships to make predictions from previously unseen data. Here, we reserve the term “prediction” for the output of models applied to novel individuals rather than to describe brain–behavior correlations^6^. Although prediction pipelines are diverse, they typically involve four primary steps: feature selection, model building, model testing, and prediction evaluation (Fig. 2). Importantly, both feature selection and model building are performed using only training data. The resulting model is then applied unaltered to data from previously unseen individuals.

*Feature selection*: Methods for feature selection, the process of identifying model predictors, fall into two broad categories: hypothesis-driven and data-driven approaches. Hypothesis-driven methods, which leverage existing knowledge to select features, are useful for testing predictions of existing scientific models. Data-driven methods rely on statistical techniques to identify the features most relevant to individual differences in behavior. These include filter methods (selecting features based relationships with behavior), wrapper methods (considering the predictive power of different feature combinations, e.g., by systematically eliminating the least predictive features from a model), and embedded methods (incorporating feature selection into model building, such as in lasso, elastic net, and ridge regression)^147^.

Both hypothesis- and data-driven approaches can incorporate predictors from multiple domains, including genetics, brain structure and function, and behavior. The developmental trajectories of these measures, such as slope, intercept, or inflection point, may also be included. Systematically removing a predictor or predictor class from a model can identify its unique contribution to predicting outcomes or behavior^124^. Although there is no theoretical limit to the number of model features, it is best practice that they not exceed the number of observations to avoid modeling noise (overfitting)^147^. Furthermore, it is important to consider the inherent tensions between interpretability, generalizability, and variance explained. While models with fewer features may be easier to interpret, models with more features may capture additional variance in behavior and better characterize complex multimodal neural phenotypes.

*Model building*: Following feature selection, the relationship between predictors and behavior is formalized with a classifier or regression model. The goal of a classifier, such as a support vector machine or logistic regression, is to make discrete predictions. In neuroimaging research, classifiers represent the vast majority of predictive models: Of all multivariate models in translational neuroimaging, 75% were built to distinguish patients from control participants, whereas <3% were used to predict continuous symptom scores^5^. Regression models, including linear and support vector regression algorithms, make continuous rather than categorical predictions, and can facilitate the development of transdiagnostic profiles of risk or resilience for psychopathology^18^. Both classifiers and regression models can be applied to cross-sectional or longitudinal data, and the latter may incorporate techniques such as growth–curve modeling to predict past or future change^148^.

*Model testing*: Model testing, or applying a predictive algorithm to test data to evaluate its generalizability, distinguishes predictive from descriptive models. The utility of out-of-sample validation for protecting against overfitting and false positives has been discussed in detail elsewhere^5,8^. Here we highlight one dimension along which potential predictive models vary: how far out of sample they generalize.

Internal validation (i.e., *k*-fold or leave-one-subject-out cross-validation) tests whether a model generalizes to novel individuals from a single data set. Although internal validation is useful for optimizing models and conferring statistical rigor when multiple data sets are not available, it may generate biased estimates of predictive power even when evaluated with permutation testing. Despite this limitation, the vast majority of predictive models in neuroimaging have been tested with internal validation alone^5^. External validation tests whether a model generalizes beyond an initial training data set to individuals from completely independent samples. Curated data sets and platforms such as OpenfMRI^101^ that encourage data and model sharing can facilitate external validation and model refinement.

*Prediction evaluation*: Methods of model evaluation depend on whether predictions are discrete or continuous. Classifier output can be evaluated with percent accuracy; sensitivity (the true positive rate, or percent of correctly identified patients) and specificity (the true negative rate, or percent of correctly identified controls); and/or the positive predictive value (percent of individuals called patients who are true patients) and negative predictive value (percent of individuals called controls who are true controls), which depend on disease prevalence. Regression model predictions can be assessed with measures such as correlation or mean-squared error^20^. In all cases it may be useful to visualize all data points to fully evaluate relationships between behavior and predicted scores or category labels.
